# Novel Polyvinyl Alcohol (PVA)/Cellulose Nanocrystal (CNC) Supramolecular Composite Hydrogels: Preparation and Application as Soil Conditioners

**DOI:** 10.3390/nano9101397

**Published:** 2019-10-01

**Authors:** Zuo Wang, Yaoke Ding, Jincheng Wang

**Affiliations:** College of Chemistry and Chemical Engineering, Shanghai University of Engineering Science, Shanghai 201620, China; wangzuo111@163.com (Z.W.); dingyaok1989@126.com (Y.D.)

**Keywords:** cellulose nanocrystal, polyvinyl alcohol, supramolecular interactions, soil conditioner, properties

## Abstract

In this work, cellulose nanocrystal (CNC) was modified by an ureido-pyrimidinone (UPy) system based on quadruple hydrogen bondings, and CNC-UPy was obtained. Then, this powder was added into polyvinyl alcohol (PVA), and PVA/CNC-UPy composite membranes and hydrogels were prepared. Fourier transform infrared spectroscopy (FTIR), X-ray diffraction (XRD), polarizing optical microscopy (POM) and particle size distribution (PSD) were used to characterize CNC-UPy. From the FTIR results, the characteristic peaks of NCO group sat 2270 cm^−1^ disappeared, indicating the successful synthesis of CNC-UPy. XRD results showed that the modification by UPy may change the structure of CNC and its degree of crystallinity was increased. PSD analysis showed that the particle size of CNC was increased and its size distribution became narrower after modification by UPy groups. The structure and properties of the composite membranes and hydrogels were studied by differential scanning calorimeter (DSC), thermogravimetric analysis (TGA) together with investigation of swelling, sustained release and self-healing performances. DSC curves depicted that the glass transition temperature, T_g_, of different PVA membranes was increased with addition of different proportions of CNC-UPy. TGA data showed that the temperature of maximum weight loss rate was increased, which illustrated the enhanced thermal stability of PVA/CNC-UPy composites. Meanwhile, it was also revealed that the PVA/CNC-UPy composite hydrogels possess good self-healing and better sustained release behavior for the soil conditioner, fulvic acid (FA).

## 1. Introduction

In recent years, many problems, such as soil salinization, nutrient and microbial imbalance, have seriously restricted the intensive development of urban agriculture and become an urgent problem to be solved in modern facility agriculture in China. Soil conditioners area kind of medicine which are used to regulate elements of soil that lose nutrients due to overuse or pollution. As a type of soil conditioner, fulvic acid (FA) may be used in regulation of plant growth, which can play an important role in drought resistance, improve plant stress resistance, increase yield and improve quality. However, this type of conditioner cannot retain nourishment with a certain amount for a certain time.

Polyvinyl alcohol (PVA) is a kind of biopolymer with good biocompatibility, good water solubility and easy processing behavior [1,2]. It is white in color and has a flaky, flocculent or powdery morphology. PVA has many excellent advantages compared with other polymer materials, such as its good hydrophilicity, which makes it play a huge role in the chemical industry, textile industry and construction field. As a kind of biopolymer, it is widely used in biomedicine and other fields. In addition, it has been applied in some agricultural and chemical fields [3,4,5]. Hydrogels area type of stretchable, compressible and cross-linked hydrophilic polymer with three-dimensional networks, adjustable water content and switchable mechanical structures, while maintaining their structural integrity during deformation [6,7]. In addition, they are considered to be powerful material platforms, providing novel ways to design soft bioelectronics and flexible energy storage/conversion devices, such as wearable devices and implantable devices, release devices and electronic skins. PVA can form a type of hydrogel, and the better hydrophilicity makes it play an important role in improvement of the adsorption and slow release of soil conditioner. However, the PVA hydrogel may exhibit low tensile properties, which are not beneficial for its subsequent applications [8,9].

Cellulose nanocrystal (CNC) is a new kind of really renewable, environmentally friendly and superior material with light weight, excellent mechanical properties, good biodegradability and renewability [10,11]. Surface treatment technology is used to change the surface environment of cellulose by using a compound with a target group to react with the active groups on its surface [12,13,14,15,16,17]. For example, Sato et al. [10] modified CNC by esterification with alkenyl succinic acid. In addition, CNChas also been modified by acetylation methods. Lee et al. [11] synthesized esterified CNCs with different degrees of substitution by reacting it with fatty acids possessing different molecular chain lengths. The modified CNC exhibited better hydrophobic properties. Huang et al. [13] prepared esterified CNC by a ball milling method. Tetrahydrofuran was used as a reaction medium. The pretreatment process of mechanical ball milling was accompanied by acetylation of the cellulose. At present, CNC is used as a reinforcing phase in synthetic polymers such as PVA, epoxy resins, polyacrylic acid, polystyrene, polycaprolactone and cellulose [18,19,20,21,22]. Li et al. [20] used photo-crosslinking technology to prepare a composite double-network hydrogel, in which poly(N-isopropylacrylamide) (PNIPPAm), polyacrylic acid (PAA),PVA and CNC were included. A series of mechanical testing results illustrated that the PVA/CNC/P (NIPPAm-co-AA) hydrogels showed good mechanical properties. Popescu et al. [21] used a solvent casting method to prepare a novel type of bio-nanocomposite films, and CNC reinforced PVA composites were thus obtained. The results exhibited that with the addition of low amounts of CNC into PVA composites, their degree of crystallinity and water sorption ability were reduced, and it was shown that the amount of hydroxyl groups in the new hydrogen bonded systems were increased. Tanpichai et al. [22] successfully prepared CNC reinforced PVA hydrogels, using glutaraldehyde (GA) as a cross-linker. The swelling ratio and thermal stability of the PVA hydrogels were not affected obviously. The creep elasticity was decreased due to the incorporation of CNC, which was ascribed to the restriction of the molecular chains. However, till now, the modification effect of CNC was not very obvious, and the sustained release effect of PVA hydrogel, especially for soil conditioners, has not been investigated.

In recent years, significant developments have been attained in supramolecular chemistry, such as self-healing materials, degradable drug nanocarriers, molecular muscles, and stimuli-responsive supramolecular gels [23,24,25,26,27,28].In addition, these interactions may be ascribed to hydrogen bonds, π-π interactions, hydrophobic interactions, and so on [29,30]. Among them, hydrogen bonding systems represent the most essential secondary interaction to construct supramolecular molecules on account of their moderate strength, high directionality, and accurate selectivity.

In this work, a quadruple hydrogen-bonding ureido-pyrimidinone (UPy) system was selected and used to modify CNC with hydroxyl groups based on a carbamate method, and quadruple hydrogen bonding was exhibited in this novel filler, called CNC-UPy. Then, a kind of PVA/CNC-UPy supramolecular composite membranes and PVA/CNC-UPy/FA hydrogels were prepared by blending PVA with the modified CNC and FA. The intermolecular interaction force was strong, making it possess better thermal stability, higher mechanical strength and sustained release behavior of the soil conditioner.

## 2. Experimental

### 2.1. Materials

Polyvinyl alcohol (PVA, 1788, AR), 6-methylisocytosine (MIC, 98%+), N-N-dimethylformamide (DMF, rectification stage), pentane (99.5%), dibutyltinlaurate (DBTDL, 95%+) and borax powder were all supplied by Shanghai Taitan Technology Co., Ltd. (Shanghai, China). Hexamethylenediisocyanate (HDI, 99%) was received from Shanghai Xilong Biochemical Technology Co., Ltd. (Shanghai, China). Cellulose nanocrystal (CNC, industrial grade) was bought from Shanghai Flash Nanotechnology Co., Ltd. (Shanghai, China).Fulvic acid (FA, 99% pure) was obtained from Hefei BASFBio technology Co. Ltd. (Anhui, China). Deionized water was obtained from Shanghai Aladdin Biochemical Technology Co., Ltd. (Shanghai, China).

### 2.2. Preparation of CNC-UPy

MIC (0.1 mol) and HDI (0.6 mol) were both added into a 250 mL flask under a nitrogen atmosphere. The reaction conditions were set at 100 °C, and the condensation under reflux was continued for 16 h. After this reaction, *n*-pentane was added, and a white solid was precipitated, washed, filtered and collected. Last, the precipitate was dried in a vacuum oven at 50 °C to obtain a white powder, UPy-NCO. CNC (1 g) was added into a 250 mL flask, using DMF as solvent. Then, UPy-NCO (2 g) was added into the above solution, and DBTDL was added as catalyst. This mixture was stirred for 14 h, and the reaction temperature was set at 120 °C. The condensation under reflux was performed under a nitrogen atmosphere. The product, CNC-UPy (UPy groups: ~30%), was obtained after washing several times with deionized water at 9000 rpm.

### 2.3. Preparation of PVA Supramolecular Composite Membranes

PVA (20 g) was added into deionized water (80 mL), and the mixture was stirred at 100 °C for 2 h until it was dissolved completely. CNC-UPy was mixed with distilled water in different proportions, and was added into the above PVA aqueous solutions under ultrasound dispersion. After stirring for 2 h, the final mixed solution was poured into the glass template, and the PVA/CNC-UPy supramolecular composite membranes were obtained.

### 2.4. Preparation of PVA Supramolecular Composite Hydrogels

First, borax powder (0.8 g) and FA (0.5 g) were dissolved in waterwith different proportions of CNC-UPy (0.01, 0.02, 0.04, 0.06, 0.1 g), and the mixtures were stirred continuously for 20 min at room temperature. Then, PVA powder (4 g) was slowly added to the above aqueous solutions. When the PVA powder was fully swelled, the solution was heated to 90 °C and stirred for 2 h. After it was completely dissolved, a uniform mixture of PVA-borax system was formed. Next, the temperature was dropped, and viscoelastic phenomena began to appear in the solution. After stirring was stopped, the solution gradually turned into the final hydrogel. A series of composite hydrogels with different proportions of CNC-UPy, called PVA/CNC-UPy/FA, were thus obtained.

### 2.5. Characterization

#### 2.5.1. Fourier Transform Infrared Spectroscopy (FTIR)

FTIR was used for structure measurement of groups using a Nicolet AVATAR 370 model spectrometer (Thermo Fisher, Waltham, MA, USA). The potassium bromide tablet method was used to analyze CNC, UPy-NCO and CNC-UPy, and the ATR total reflection method was applied to test the structure of the different PVA composites. The number of internal reflections of the ATR was 10 times. The scanning frequency was set sixty four times, and the resolution was 4 cm^−1^.

#### 2.5.2. X-ray Diffraction (XRD)

XRD was adopted to test the crystal structure of different materials, using a D2 PHASER diffractometer (Bruker, Karlsruhe, Germany). The X-ray beam was the CuKa (λ = 0.154 nm) radiation filtered by nickel, which was operated at 50 kV. The scanning range was from 5 to 80°, and the scanning rate was 2°/min.

#### 2.5.3. Scanning Electron Microscopy (SEM) and Polarizing Microscope (POM)

SEM was tested with SU8010 200 kV equipment (Hitachi, Tokyo, Japan). Photographs were taken at 5 kV potential. The samples need to be gold sprayed before testing. POM was applied to take photographs with a XPF-300 instrument (Tianxing, Shanghai, China) using a magnification of 400–1000 times.

#### 2.5.4. Particle Size Distribution (PSD)

PSD was measured by an URT 210-C particle size analyzer (Ziyue, Shanghai, China). Samples (0.05 g) were dispersed in 100 mL of deionized water by ultrasound. The refractive index is n 20/D 1.504.

#### 2.5.5. Differential Scanning Calometry (DSC)

DSC was performed on a Q600 SDT differential thermal scanner (TA Instruments, New Castle, DE, U.S.A.). The temperature range used was from room temperature to 120 °C. The temperature was raised and lowered at a rate of 5 °C/min in a nitrogen atmosphere.

#### 2.5.6. Thermogravimetric Analysis (TGA)

TGA was carried out using the TA Q600 SDT instrument (TA Instruments, New Castle, DE, U.S.A.). Thermal stability of PVA and PVA/CNC-UPy composites was evaluated in nitrogen atmosphere with a heating rate of 20 ˚C/min in a temperature range from 25 to 800 °C.

#### 2.5.7. Swelling Behavior Analysis

The PVA/CNC-UPy/FA composite hydrogels with different proportions of CNC-UPy were freeze-dried and weighed. Their initial weight was recorded, and then they were placed into deionized water. After a certain interval of time, such as 10, 40, 100, 220, and 340 min, the hydrogels were removed from the deionized water with tweezers. Then, the moisture was gently wiped off on their surface with absorbent paper. The swelling ratio was calculated using following formula:

(1) $$Swelling\;ratio\;\left(\%\right)=\frac{\mathrm{Swelling}\;\mathrm{weight}}{\mathrm{Initial}\;\mathrm{weight}}\times 100\%$$

#### 2.5.8. Sustained Release Behavior Analysis

PVA/CNC-UPy/FA composite hydrogel (5 g) was added into a dialysis tube which was filled with 25 mL of deionized water. An ultraviolet spectrophotometer was used to measure the amount of FA released. The concentration of FA was obtained based on standard curves, which were originated from the standard liquid with gradient concentration. The release performance of FA from the composite hydrogel was tested in a temperature range from 0 to 350 h. Three times were measured for each sample, and thus the reliability of the experiments can be realized.

#### 2.5.9. Self-healing Performance Analysis

A PVA/CNC-UPy/FA composite hydrogel was chosen and divided into three sections. They were dyed and named A, B and C. The three parts of the hydrogels were then put next to each other, and we waited for 3 min. Afterwards, the whole hydrogel composed of the above three parts was clamped on two sides for simple stretching, and its junction broken status and color changes wereobserved.

## 3. Results and Discussion

### 3.1. Preparation Mechanism of PVA Supramolecular Composite Hydrogels for Soil Conditioner

**Scheme 1:**
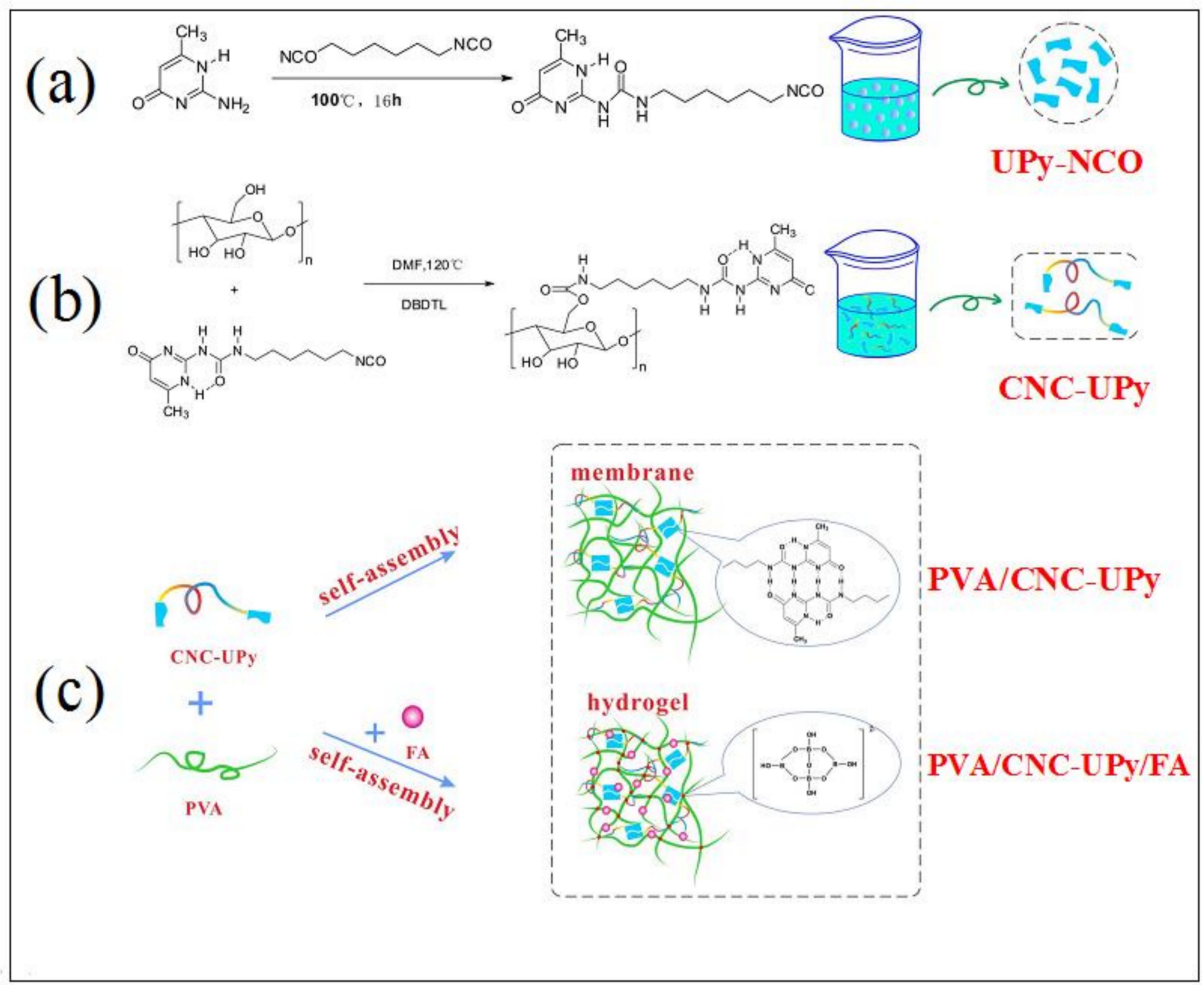
(**a**) Synthetic process of UPy-NCO; (**b**) preparation of CNC-UPy; (**c**) preparation of PVA/CNC-UPy composite membrane and PVA/CNC-UPy/FA composite hydrogel.

Scheme 1a presents the preparation process of the substituted urea, UPy-NCO, by the reaction between –NCO groups and the –NH_2_ in 6-methylisocytosine. The –NCO of UPy-NCO may react with –OH on CNC to produce aminomethyl esters, and thus CNC-UPy can be obtained (Scheme 1b). As shown in Scheme 1c, when CNC-UPy and PVA aqueous solutions were combined and self-assembled, hydrogen bonds may be formed between the –NH/–OH in CNC-UPy and –OH in PVA, and thus PVA/CNC-UPy composite membranes may be obtained after drying. PVA aqueous solution, CNC-UPy, and FA were combined with borax, and a composite hydrogel was formed from the crosslinked PVA molecules. Borax in this experiment may generate boric acid when dissolved in water. It can acquire negatively charges from OH^−^ in aqueous solution, and is then hydrolyzed into B(OH)_4_^−^. These ions, on the one hand, may take condensation reaction with hydroxyl groups in PVA molecules, and on the other hand, hydrogen bonds may be formed. Thus, the PVA molecules may be cross linked and self-assembled together to form a composite hydrogel, PVA/CNC-UPy/FA.

### 3.2. Analysis of CNC-UPy Crystals.

**Figure 1:**
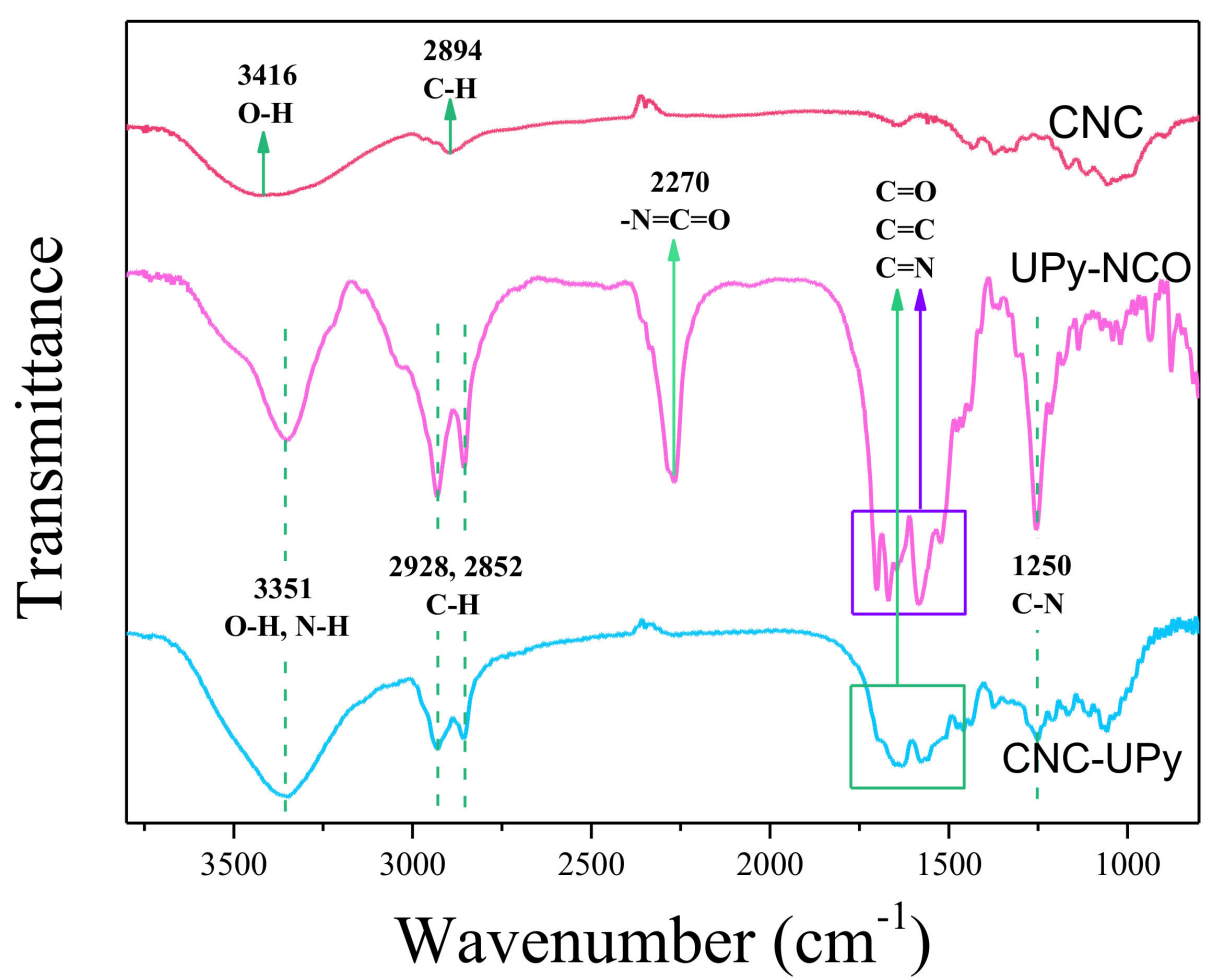
ATR-FTIR spectra of CNC, UPy-NCO and CNC-UPy.

The FTIR spectra of CNC, UPy-NCO and CNC-UPy are illustrated in Figure 1. In the curve of CNC, the broad peaks between 3000 and 3500 cm^−1^ were ascribed to -OH group absorption peaks, and the peaks at 2894 cm^−1^ resulted from C–H absorptions. In the curve of UPy-NCO, the characteristic peak at 2270 cm^−1^ was ascribed to N=C=O bonds. Moreover, the peak at 3351 cm^−1^ was ascribed to –NH groups, and the stretching absorptions at 1500–1700 cm^−1^ resulted from double bonds such as C=C, C=O, and C=N groups in the corresponding structure. In the curve of CNC-UPy, the characteristic absorption peaks of C–O in cellulose were observed at 1050 cm^−1^ and these did not appear in the UPy-NCO spectra. These characteristic peaks still existed in CNC-UPy traces, indicating that the structure of the modified nanocellulose was not destroyed. Through a carbamide esterification reaction, the –NCO groups in UPy-NCO may react with –OH groups on the primary carbon at the surface of nanocellulose, and thus CNC-UPy was successfully prepared. Moreover, the characteristic peak at 2270 cm^−1^ of N=C=O disappeared in the CNC-UPy spectrumand the peak at 3351 cm^−1^ for –NH groups became stronger. This indicated that the carbonate esterification reaction took place between –NCO of UPy-NCO and –OH in CNC, which resulted in the formation of urethane bonds. In a word, the above infrared spectrum analysis showed that UPy-NCO had reacted with nanocellulose and the UPy groups were successfully grafted onto the surface of CNC. [31,32]

**Figure 2:**
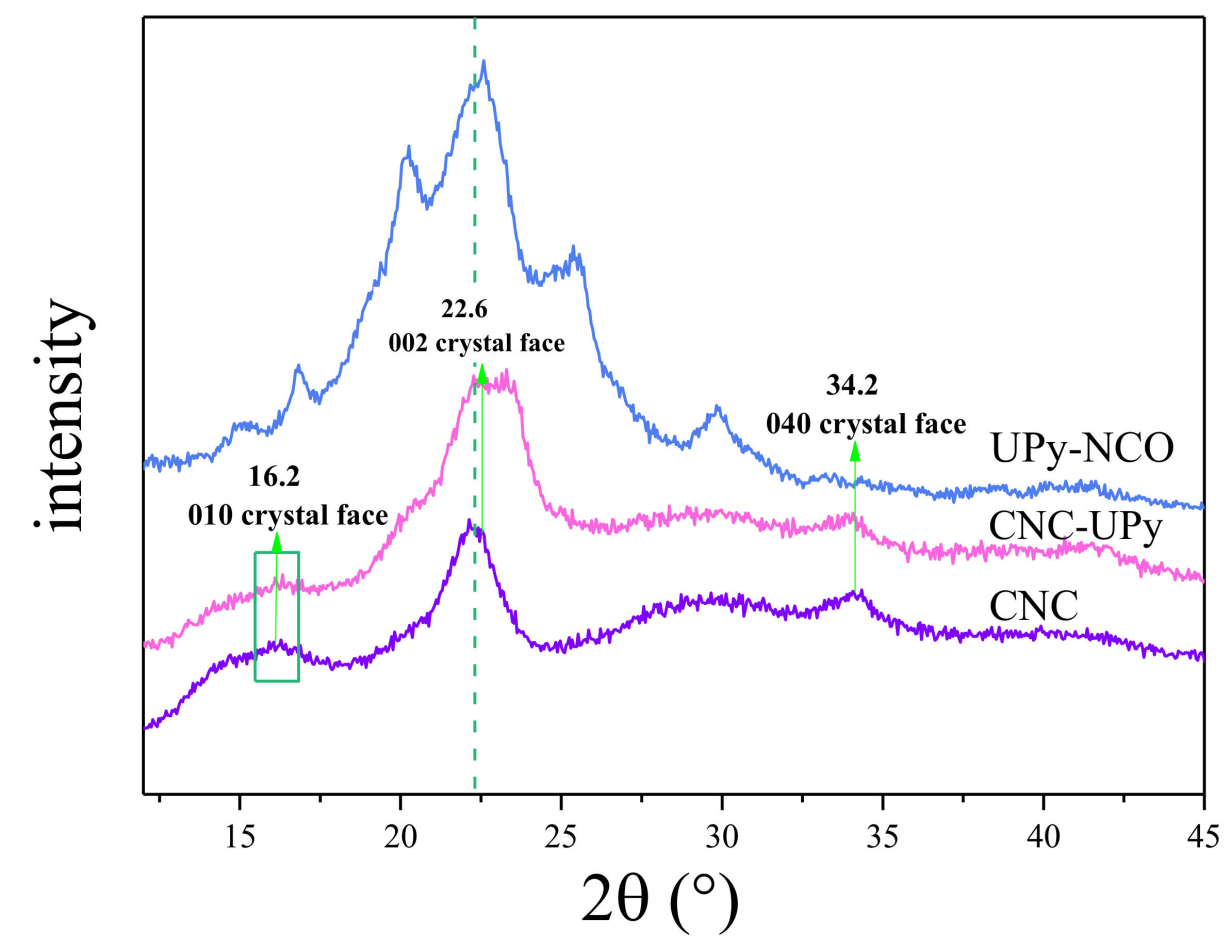
XRD curves of CNC, UPy-NCO and CNC-UPy.

XRD was used to analyze the crystal structure of CNC, UPy-NCO, and CNC-UPy (Figure 2). From the curves of CNC and CNC-UPy, the inherent crystal structure of cellulose was reflected. The characteristic peaks of cellulose crystal structure were both exhibited and located at 2θ = 16.2°, 22.6°, and 34.2°, respectively. These were corresponded to (010), (002) and (040) crystal planes of cellulose crystal. Therefore, it showed that the crystal structure of CNC-UPy had not been changed after modification of CNC. However, due to the addition of UPy-NCO, the crystallinity of CNC was improved. It can be seen that the diffraction peak area of CNC-UPy was larger than that of CNC at the same position. This may be due to the introduction of UPy-NCO groups, which can increase the number of hydrogen bonds between nanocellulose molecules. These hydrogen bonds can improve the crystallinity and the corresponding tensile strength of the composites. [33,34]

**Figure 3:**
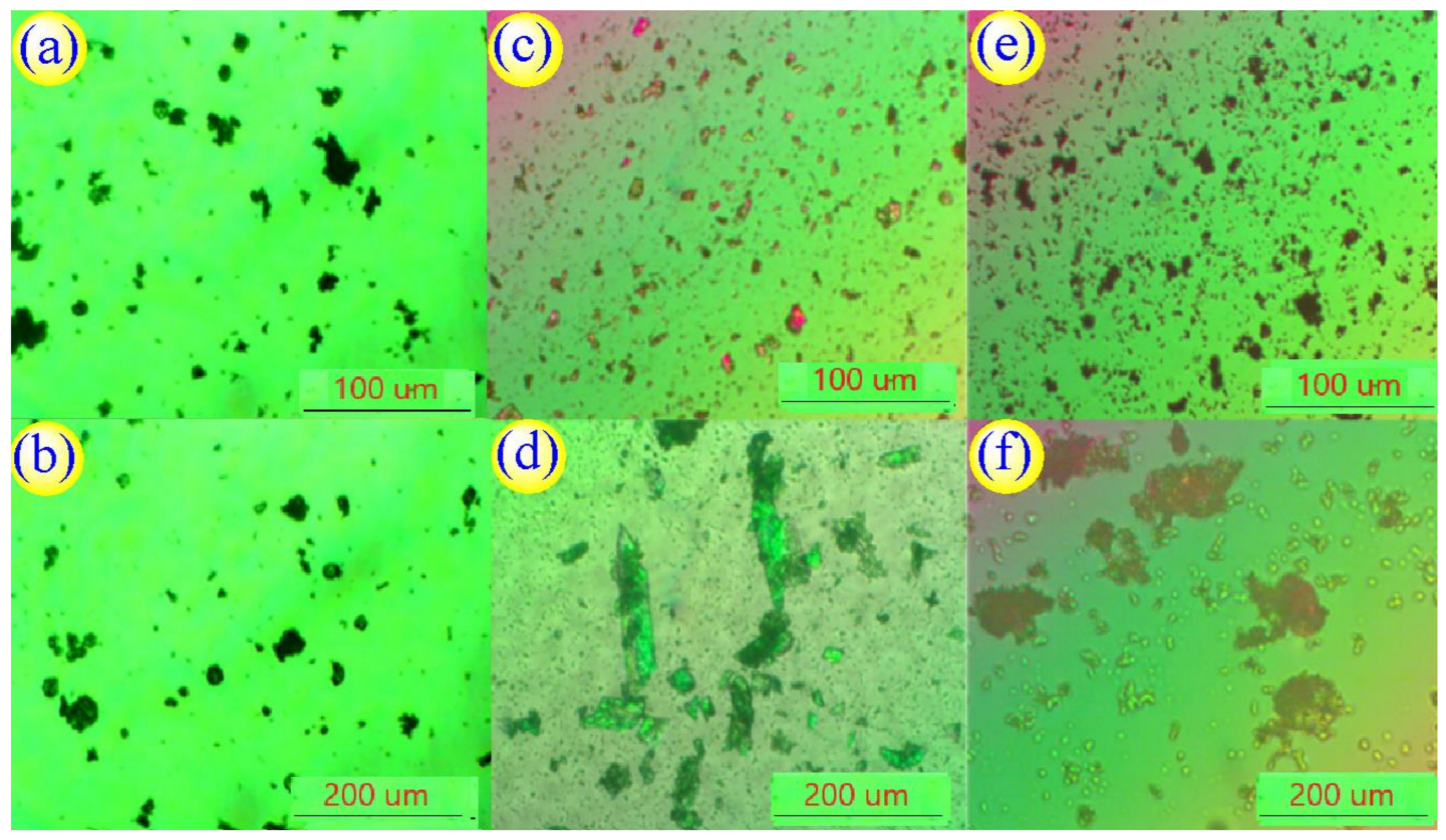
POMimages of (**a**,**b**) CNC; (**c**,**d**)UPy-NCO; (**e**,**f**)CNC-UPy.

The overall morphology of CNC, UPy-NCO and CNC-UPy was observed under POM, as shown in Figure 3. Among them, the magnification of (a), (c), (e) was 100 times, and the magnification of (b), (d), (f) was 200 times. It can be seen that CNC, (a) and (b), was a type of rod crystal, and UPy-NCO, (e) and (f), was a type of block crystal. Compared to the original CNC, the modified nanocellulose was coated with UPy-NCO. The size of CNC-UPy was large, and its dispersion was not very good [35].

**Figure 4:**
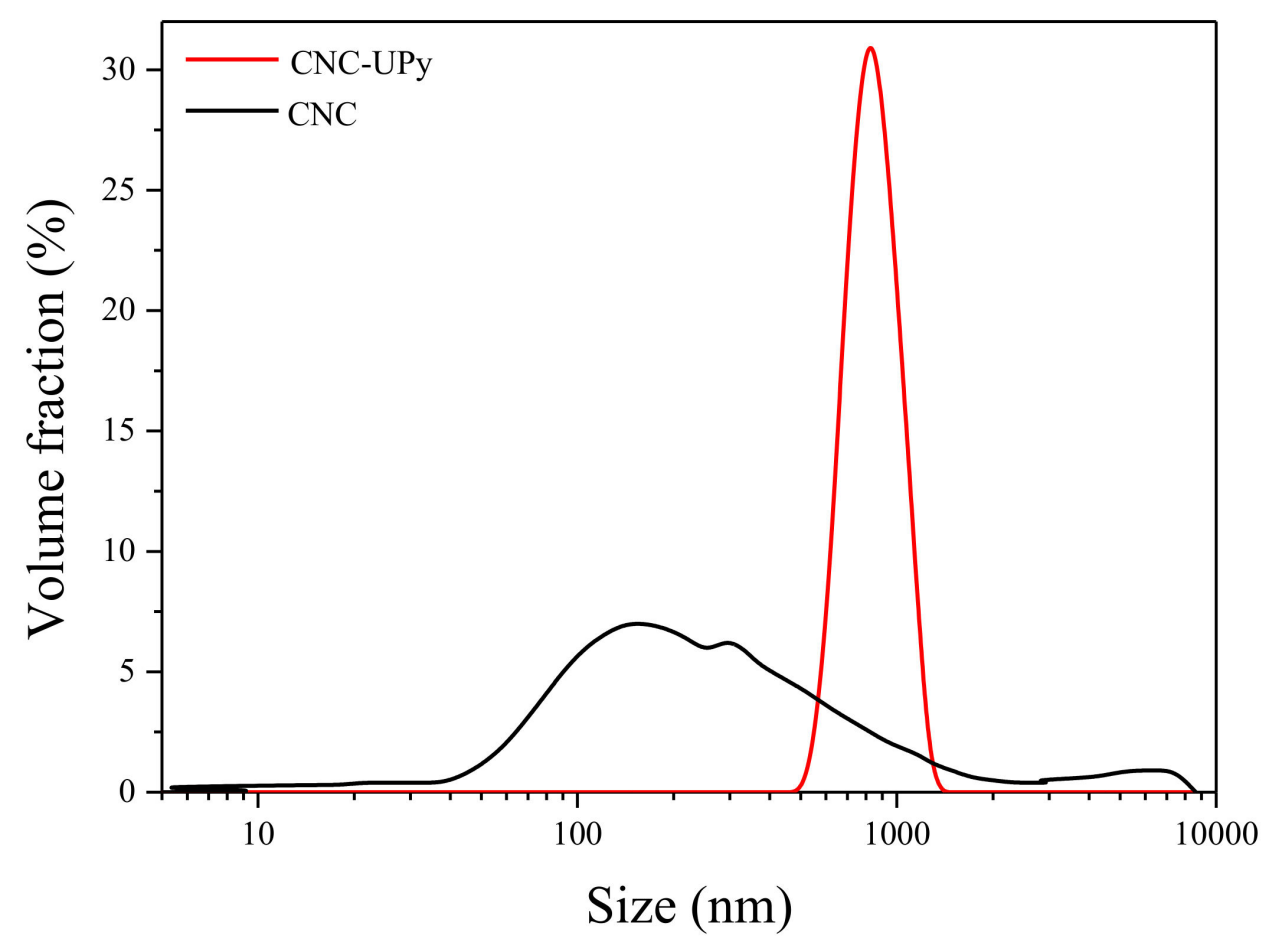
PSDcurves of CNC and CNC-UPy.

Figure 4 shows PSD curves of CNC and CNC-UPy. It can be seen that the particle size distribution of CNC was relatively wide, and the largest volume fraction was focused around 100–500 nm. However, after modification by UPy, the particle size of CNC-UPy was increased to about 600–1000 nm, and its distribution became relatively narrow. This result illustrated that the modification method was beneficial for the improvement of size uniform together with the crystallization effect of the modified material.

### 3.3. Analysis of PVA/CNC-UPy Composite Membranes

**Figure 5:**
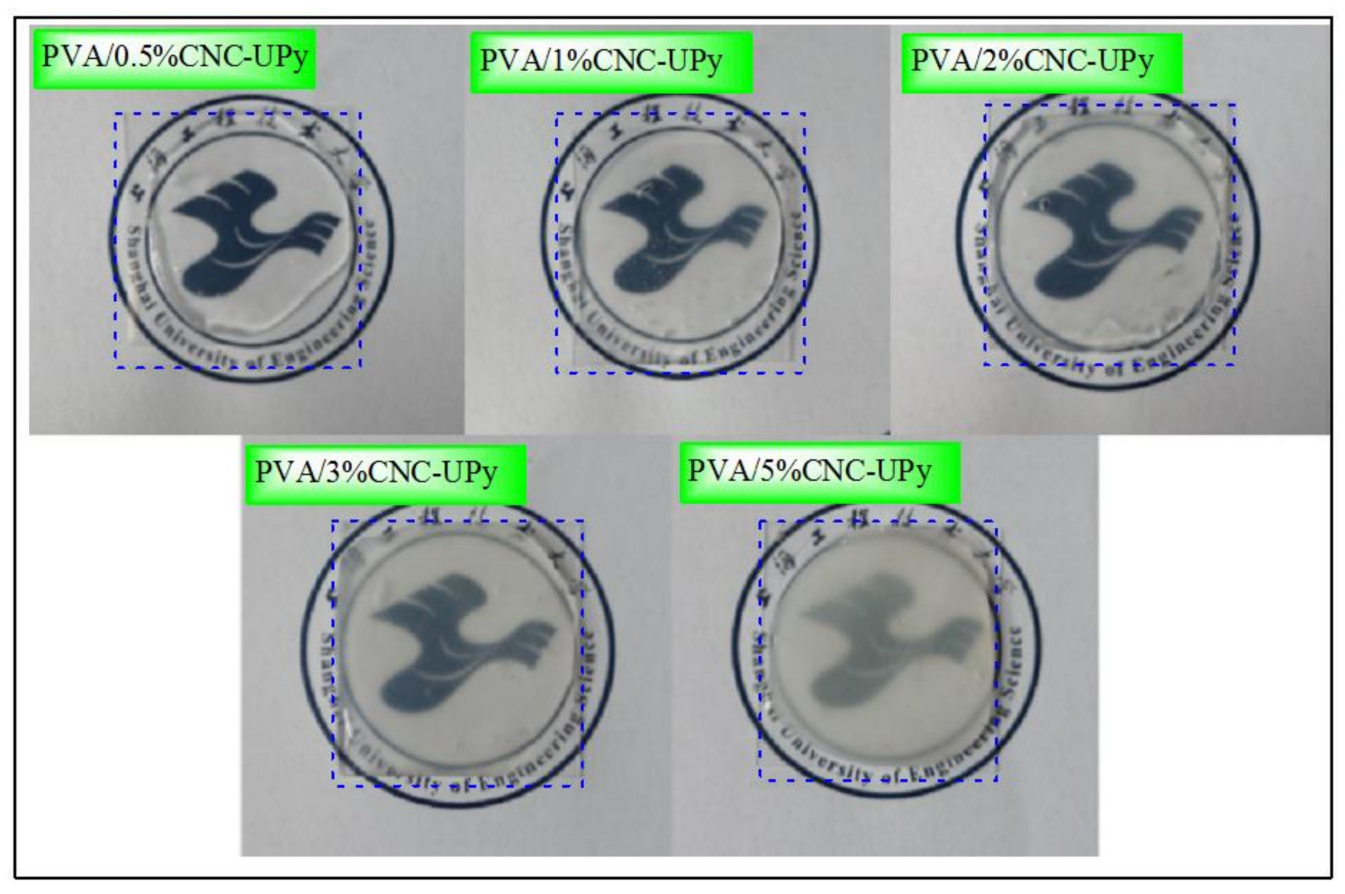
Photos of different PVA composite membranes.

Photos of PVA and different PVA composite membranes are presented in Figure 5. It can be seen that with the increase of CNC-UPy ratio, the color became whiter and the transparency became lower and lower. It was also shown that the introduction of hydrogen bonds can make these composite membranes possess some tensile properties.

**Figure 6:**
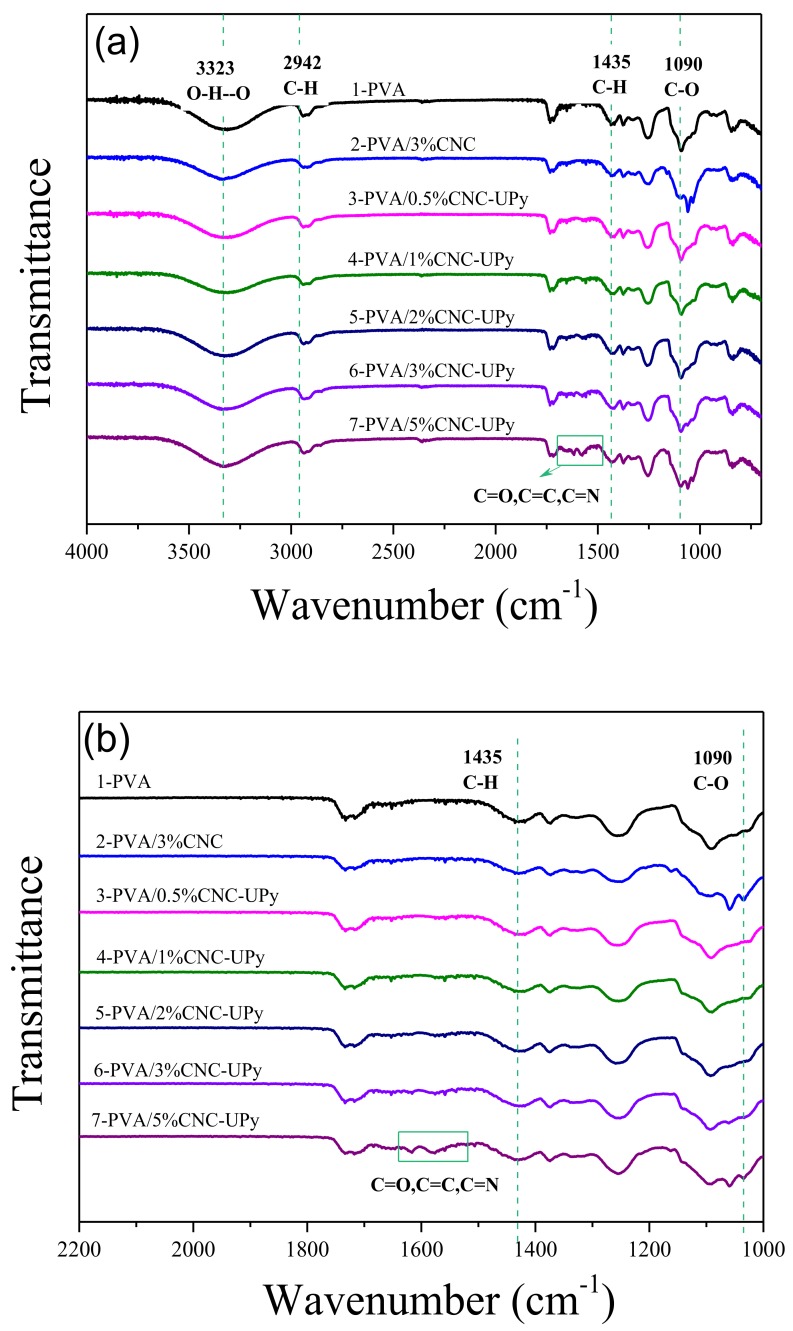
ATR-FTIR spectra of different PVA composite membranes. (**a**) Whole wavenumber ranges, (**b**) local zoom.

The FTIR spectra of PVA/CNC-UPy composite membranes with different proportions of CNC-UPy were compared and analyzed, as shown in Figure 6. In PVA, the stretching vibration of characteristic peaks of -OH in PVA molecules was located at 3323 cm^−1^, and the characteristic bands of C-H and C-O bonds were at 2942, 1435 and 1090 cm^−1^, respectively (Figure 6a). With the addition of different amounts of CNC-UPy, the curves of different PVA composite membranes were similar to those of pure PVA. In these curves, the characteristic peaks of CNC-UPy were also revealed, and some characteristic peaks appeared at 1500–1750 cm^−1^ (Figure 6b). These were resulted from the stretching vibrations of the double bonds in UPy-NCO, such as C=O, C=C and C=N. [36]

**Figure 7:**
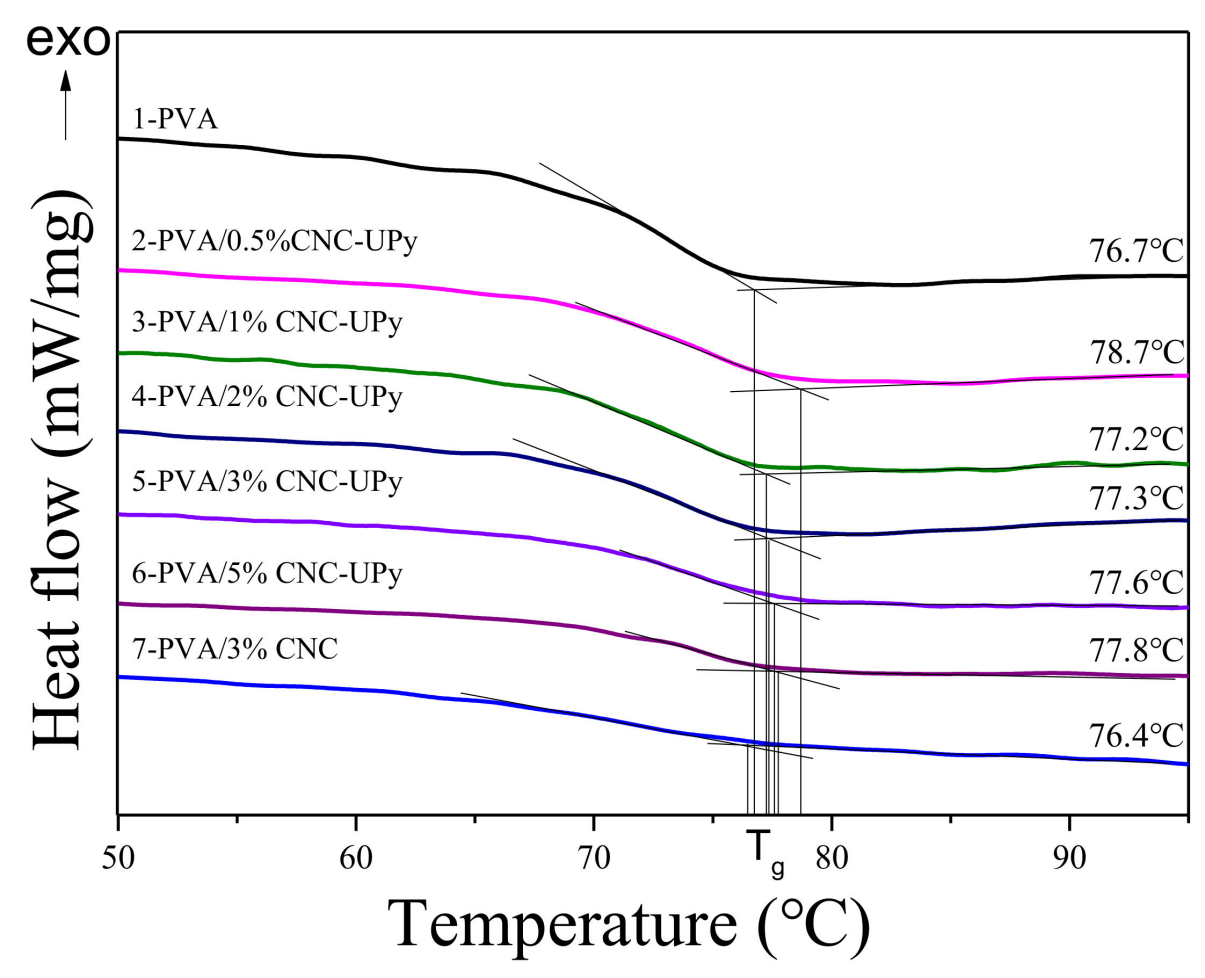
DSC curves of different PVA composite membranes.

DSC was used to analyze the glass transition temperature, T_g_, of different PVA composite membranes (Figure 7). With addition of different proportions of CNC-UPy, T_g_ of different PVA membranes was increased. On the one hand, this was attributed to the stability of CNC-UPy crystal structure and the excellent properties of CNC in various aspects. On the other hand, it also showed that there were multiple hydrogen bonds between CNC-UPy, which enhanced the binding force between PVA molecules. The addition of 0.5% CNC-UPy can increase T_g_ from 76.7 °C for pure PVA to 78.7 °C, which indicated that a small amount of CNC-UPy can improve the thermal stability of the composite membranes. Moreover, after making a comparison between PVA/3%CNC and PVA/3%CNC-UPy, it was found that T_g_ was increased from 76.4 to 77.6 °C. This was attributed to the reason that the crystallinity of CNC-UPy was higher than that of CNC, which fully demonstrated that the degree of crystallinity of the composite can be greatly increased by introducing the ADD-type quadruple hydrogen bonding systems. This property was attributed to the stronger hydrogen bond based supramolecular interactions between CNC-UPy crystals and the macromolecular chains of the PVA matrix, which improved the crosslinking degree together with T_g_ value of the composite membrane. [5,37]

**Figure 8:**
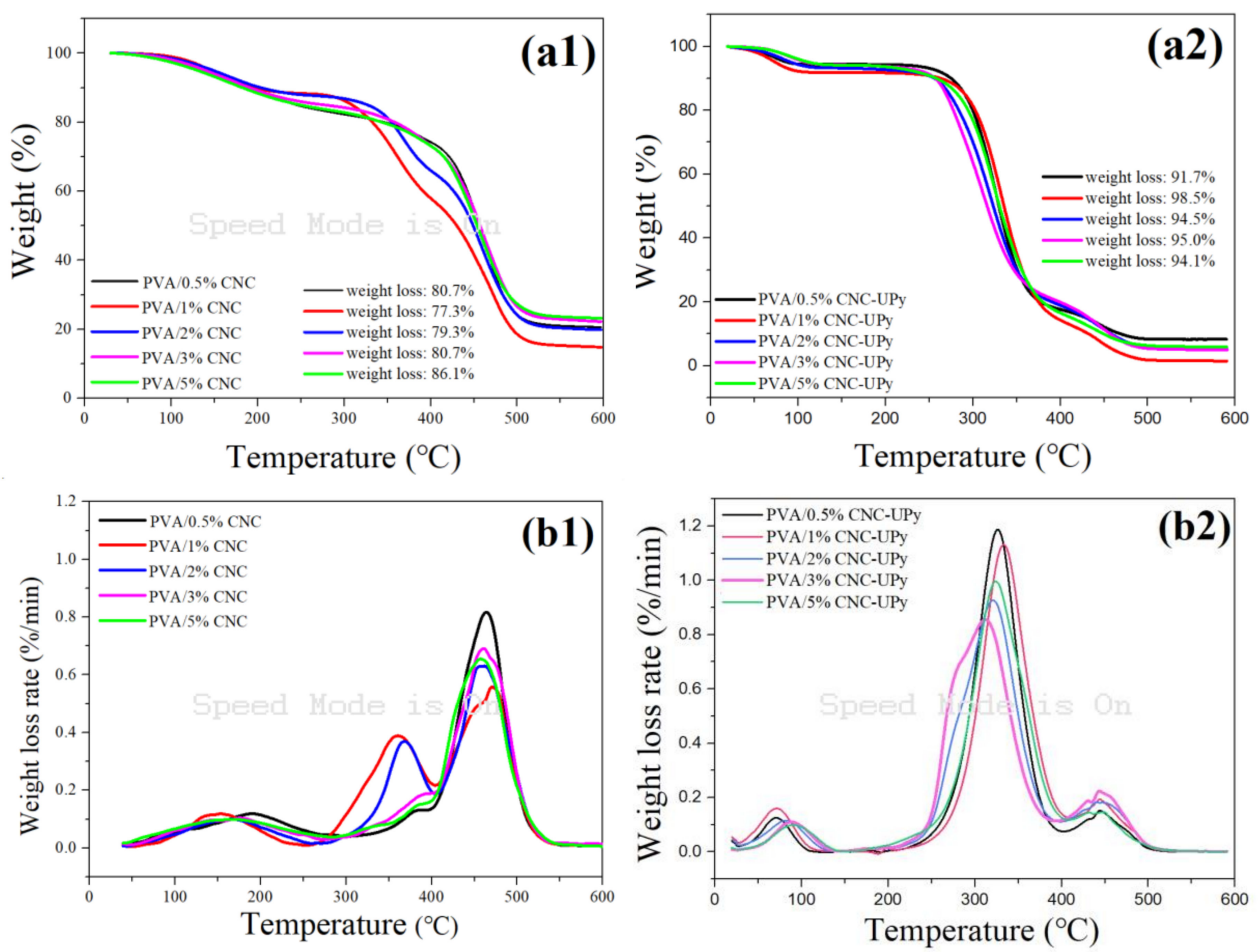
TGA (**a1,2**) and DTG (**b1,2**) curves of different PVA composite membranes.

TGA and DTG curves of different PVA composite membranes are exhibited in Figure 8. Thethermal degradation process, corresponding decomposition temperatures and weight loss of each composite were observed. From Figure 8a1,b1, the PVA/0.5%CNC presented higher thermal stability, especially in the temperature range between 280 and 430 °C. As can be seen from Figure 8a2, compared to other composites, the PVA/0.5%CNC-UPy also exhibited better thermal stability, especially in the temperature range between 150 and 300 °C. From Figure 8b2, the DTG curves of five kinds of PVA membranes, it can be seen that their mass loss can be divided into three stages. The first stage was attributed to the volatilization of water adsorbed in the composites before 100 °C, and the second stage started at about 310 °C. This was caused by the absorbed moisture and the degradation of the side chains of PVA molecules. And the third stage began at about 450 °C, which was resulted from the degradation of the main chains of PVA molecules and the combustion of carbon materials [38,39]. In addition, PVA/3%CNC-UPy composite membranes presented the lowest degradation degree compared to other composites. After making a comparison between Figure 8a1,a2 and b1,b2, it can be seen that the modification of UPy can improve the thermal stability of PVA between 100 and 250 °C. However, the thermal stability was decreased when the temperature was higher than 300 °C.

### 3.4. Analysis of PVA/CNC-UPy Composite Hydrogels

**Figure 9:**
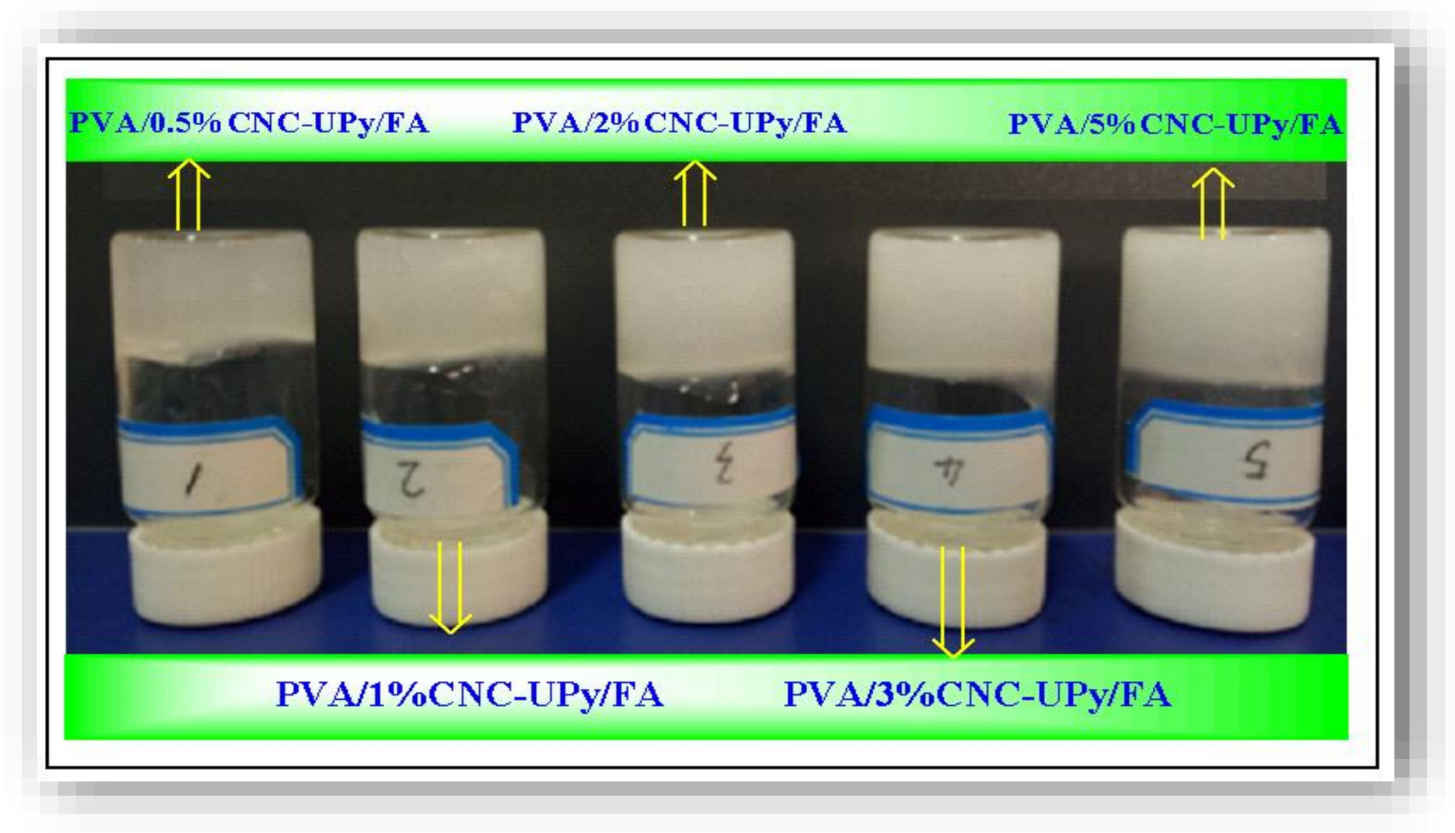
Photos of different PVA/CNC-UPy/FA composite hydrogels.

Photos of different proportions of PVA/CNC-UPy/FA composite hydrogels were obtained and are illustrated in Figure 9. It can be seen that the prepared hydrogel had no fluidity, and with the increase of CNC-UPy ratio, the color became whiter and the transparency became lower and lower.

**Figure 10:**
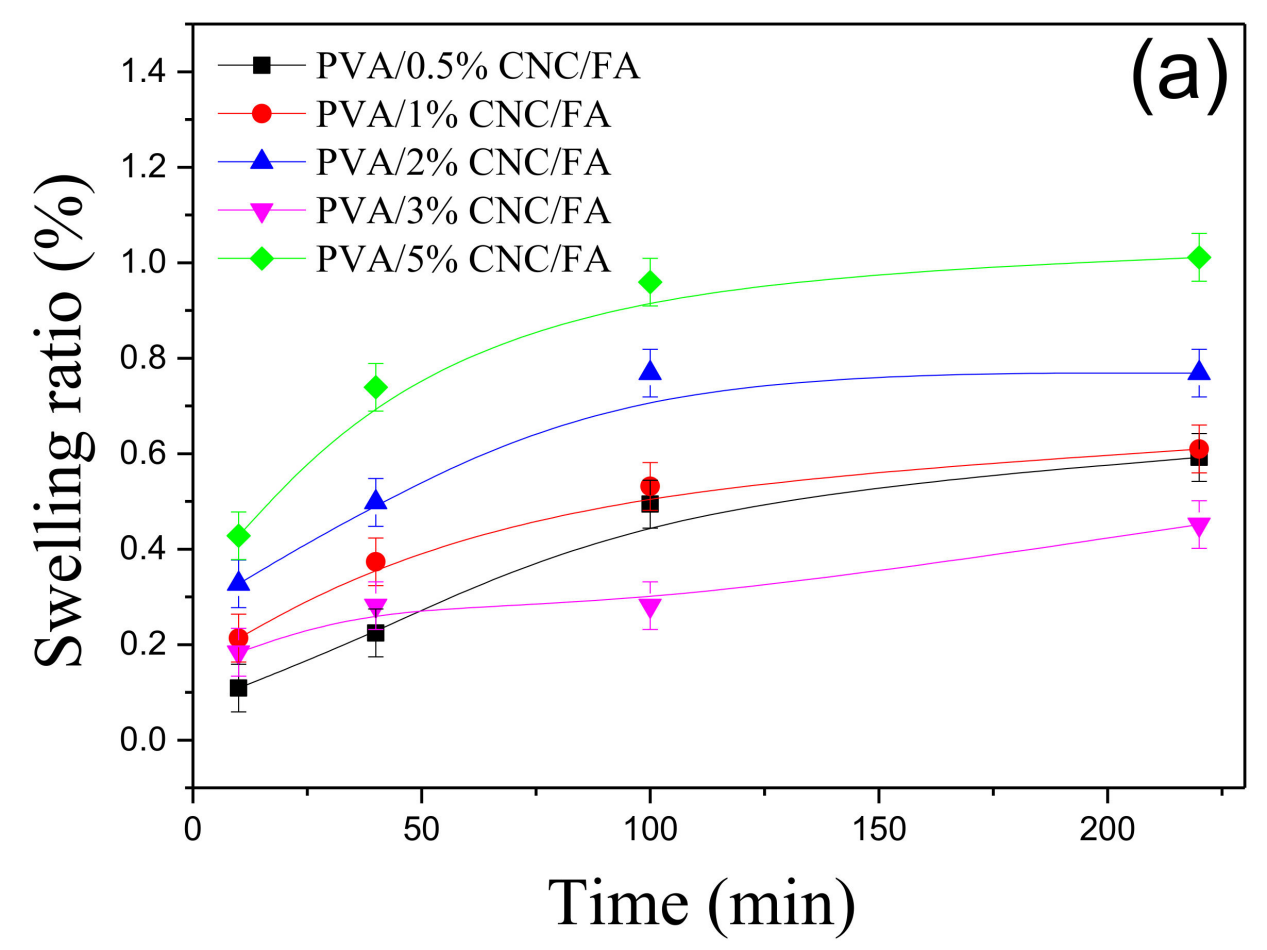

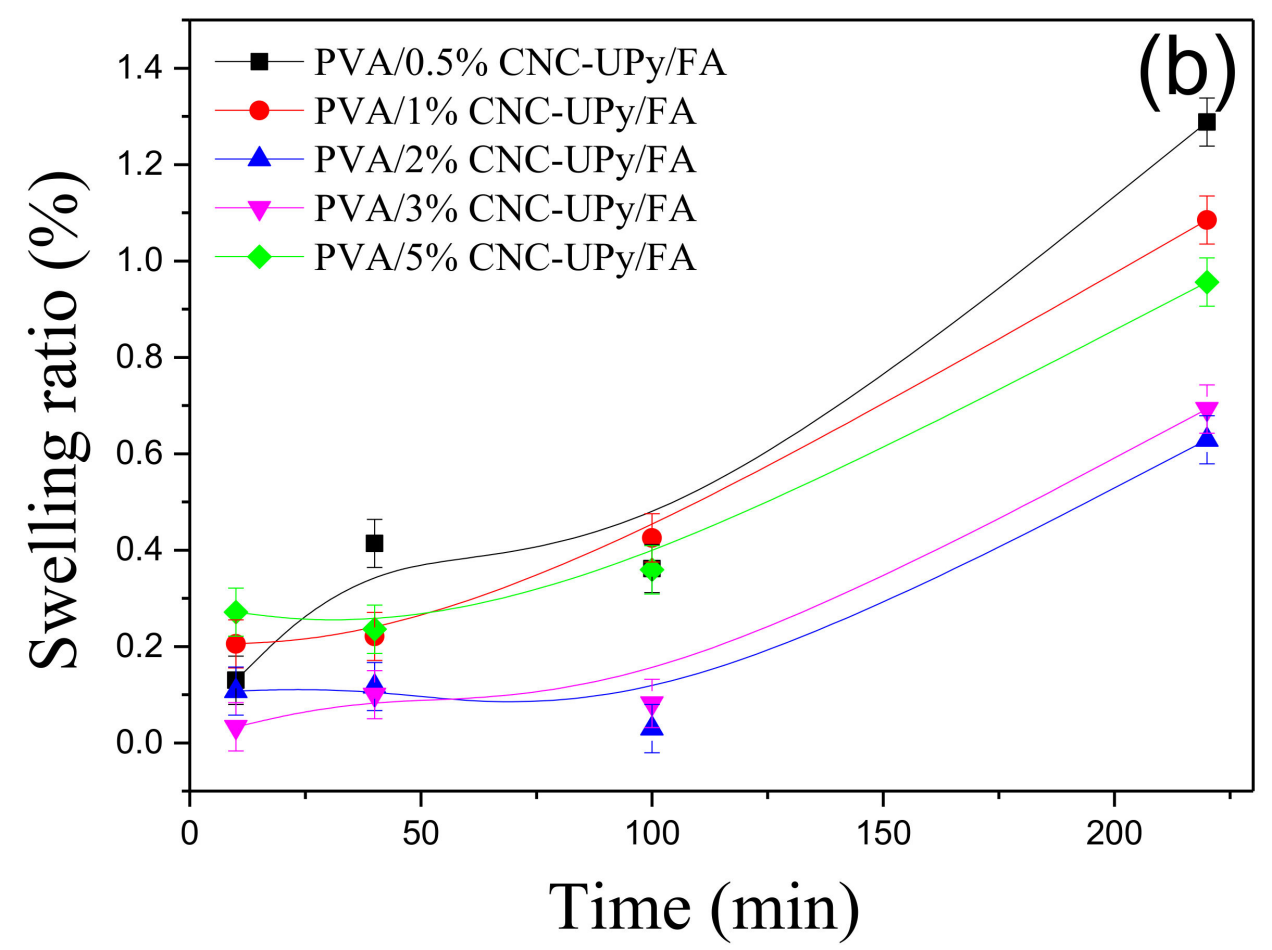
Swelling ratio of different PVA composite hydrogels (**a**) PVA/CNC/FA, (**b**) PVA/CNC-UPy/FA.

Figure 10 shows the swelling ratio curves of PVA composite hydrogels with different proportions of CNC and CNC-UPy together with FA. As can be seen, an increasing trend was evident in the above curves. PVA was easily soluble in water, and its swelling properties were relatively poor. In Figure 10a, during the whole time range, the swelling ratio of PVA/5%CNC/FA was the largest and the swelling property was the best. This illustrated that this hydrogel can absorb more water which showed less FA was adsorbed in the matrix. More amount of CNC existed in the matrix and this may cause bad compatibility and large holes. These holes have the least adsorption force with FA. Thus, more water can be absorbed and the swelling ratio was improved. In Figure 10b, the swelling ratio of the hydrogels, PVA/0.5%CNC-UPy/FA, was the largest between 100–200 min, and the swelling property was the best. That is, on one hand, the least amount of FA existed in this composite hydrogel; on the other hand, it possessed many small holes and can absorb a lot of water due to the weak binding forces of the hydrogen bonds. As for PVA/2%CNC-UPy/FA and PVA/3%CNC-UPy/FA, the CNC-UPy powder was dispersed uniformly in PVA matrices. In addition, more hydrogen bonds were formed in the CNC powders due to the UPy groups, and thus the adsorption ability may be improved and more amount of FA can be adsorbed. This may lead to the less water absorbed in these composite hydrogels. However, when excessive modified CNC-UPy was added, that is, PVA/5%CNC-UPy/FA, will occupy some holes and less space was remained, and these were not beneficial to the swelling and adsorption properties of the hydrogels. [40]

**Figure 11:**
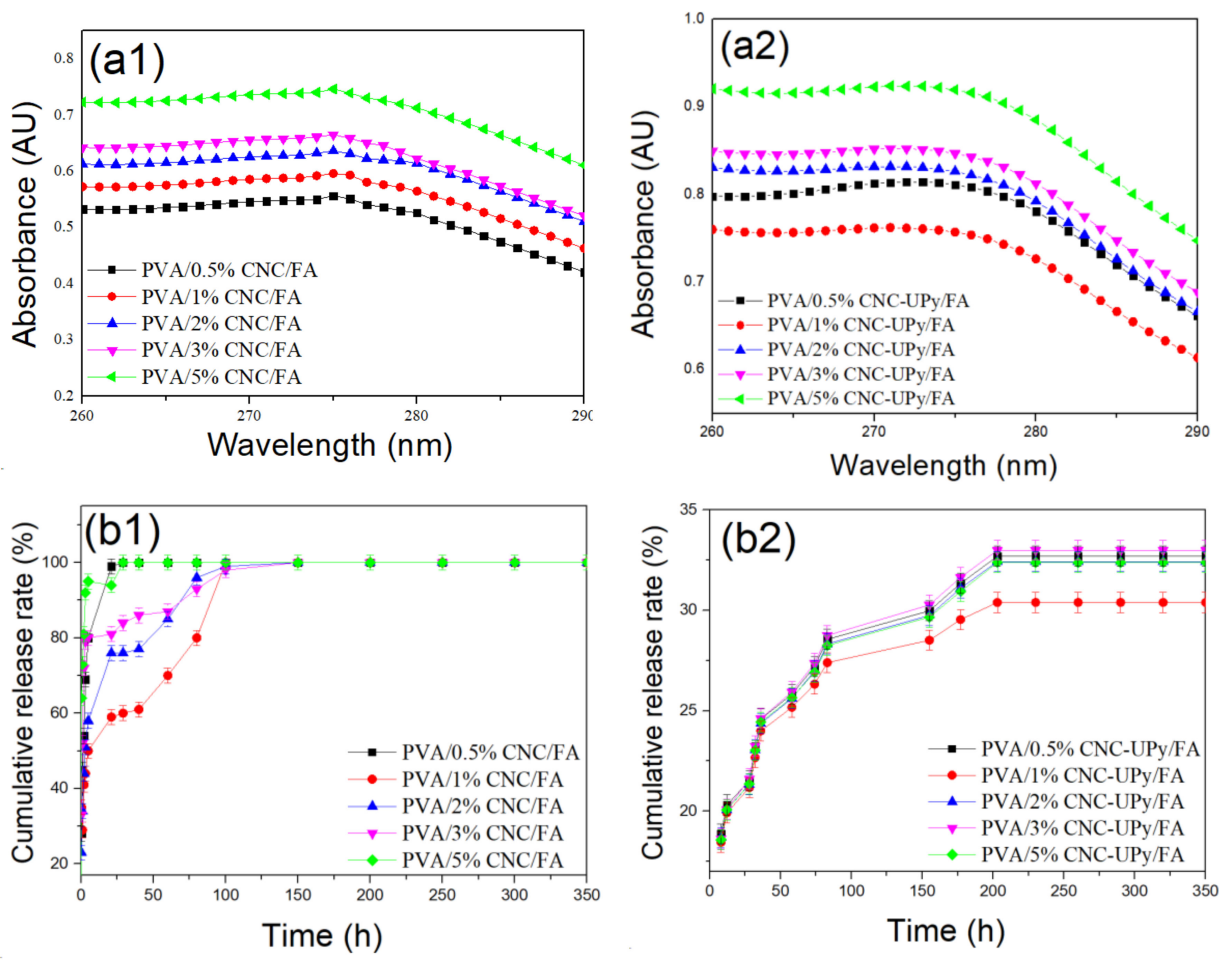
(**a1,2**) UV spectra of different PVA composite hydrogels at 24 h,(**b1,2**) sustained release behavior of PVA composite hydrogels.

**Figure 12:**
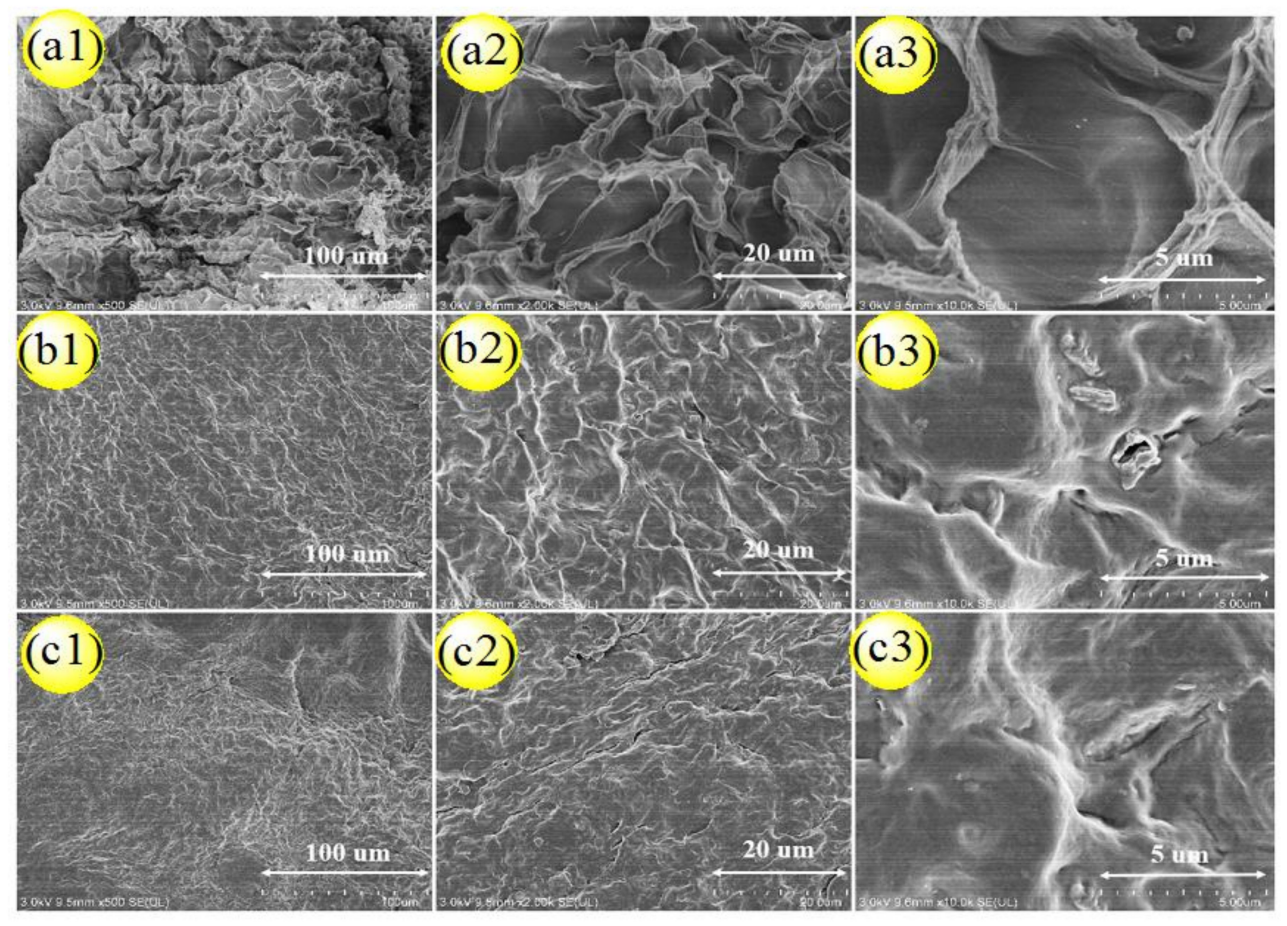
SEM images (**a1–3**)0.5%CNC-UPy/PVA, (**b1–3**)3% CNC-UPy/PVA, (**c1–3**) 5% CNC-UPy/PVA.

Figure 11 presents the absorbance change of UV spectra and sustained release behavior of different PVA composite hydrogels. From Figure 11a1,b1, during the first 24 h, the release rate of FA out of the composite hydrogels was almost in direct correlation with the CNC content except for PVA/0.5%CNC/FA. The release rate of PVA/5%CNC/FA was the highest and it exhibited a sudden release phenomenon. This was ascribed to the presence of the least amount of FA together with the lack of adsorption behavior in this hydrogel. However, as shown in Figure 11a2,b2, an obvious slow and sustained release phenomenon appeared for the composite hydrogels containing UPy modified CNC. The improved absorbance of FA was ascribable to the increased interactions between –NH in CNC-UPy and -COO in FA by hydrogen bonding. The content of FA loaded in PVA/3%CNC-UPy/FA increased rapidly during the first 24 h, and its CRR was 24.8%. The concentration difference was the main reason for the spontaneous release performance. Moreover, no obvious sudden release phenomenon appeared after 200 h, and thus sustained release of FA was achieved. The PVA/3%CNC-UPy/FA exhibited better release behavior than that of other composites, and this illustrated that an appropriate amount of hydrogen bonding is beneficial for the storage and release of FA. Figure 12 presented SEM images of surface morphology of PVA/CNC-UPy/FA composite hydrogels. It can be seen from the pictures that the hydrogel had a network structure, and it became denser when the content of CNC-UPy was increased to 3% and 5%. The UPy-modified nanocellulose had more tetra hydrogen bonds, which were stronger than those of nanocellulose itself, and this may be the reason for the improved sustained release performance for these composite hydrogels.

**Figure 13:**
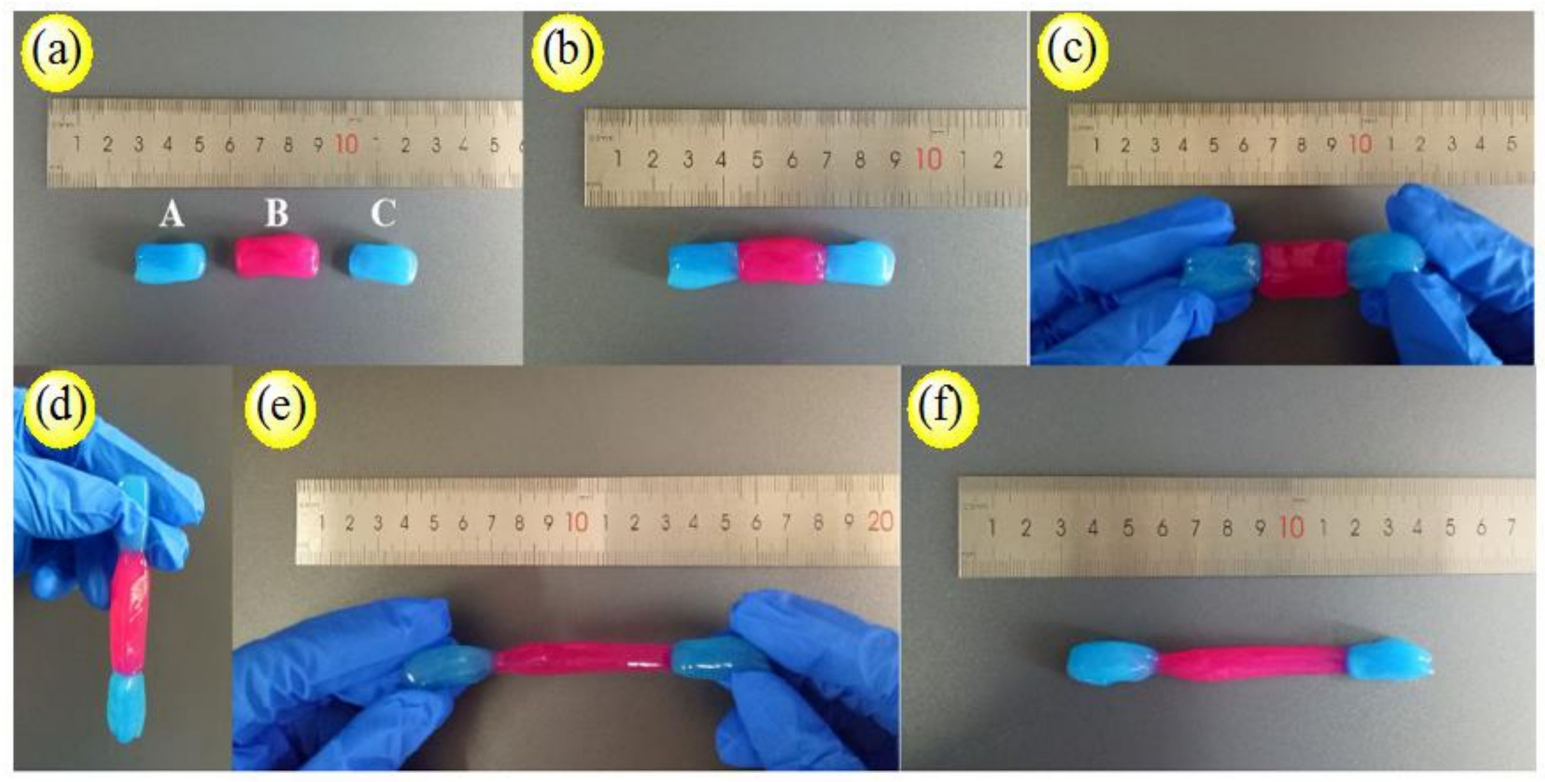
Behavior of PVA/CNC-UPy/FA composite hydrogels (**a**) three colors of hydrogelswere prepared, (**b**) three different hydrogels were placed together, (**c**) three parts of the hydrogels were bonded, (**d**) the healed hydrogels cannot be separated, (**e**) the ends of hydrogels were stretched and they grew longer, (**f**) two colors permeated each other.

The self-healing behaviors of the hydrogels is shown in Figure 13.Here, three colors of hydrogels, A, B, C, were prepared (Figure 13a). Then, three different hydrogels were placed together (Figure 13b). After 1–3 min, three parts of the hydrogels were bonded together (Figure 13c). They are not separated directly (Figure 13d). The ends of hydrogels were stretched and found that they grew longer and were not easily broken (Figure 13e). Instead, it can be seen that the two colors may permeate each other, and the color also became deeper (Figure 13f). It can be concluded that hydrogels possessed good self-healing properties. The reason was that the interactions between PVA molecules and modified cellulose particles were non-covalent hydrogen bonds. These bonds are reversible, allowing the hydrogels to self-heal through hydrogen bonding after fracture [41,42].

## 4. Conclusions

In this work, a UPy system based on the quadruple hydrogen bonding method was used to modify CNC with hydroxyl groups, and thus CNC-UPy was prepared. Then, different amounts of CNC-UPy were added to PVA aqueous solutions to prepare composite membranes and hydrogels.

FTIR analysis illustrated that the characteristic peaks of -NCO at 2270 cm^−1^ disappeared, indicating the successful synthesis of CNC-UPy. XRD results showed that CNC-UPy did not change the structure of cellulose and may improve its crystallinity. PDS analysis revealed that after modification by UPy, the particle size of CNC-UPy was increased from about 100–500 nm to about 600–1000 nm, and its distribution became relatively narrow. TGA data exhibited that the thermal stability of PVA/CNC-UPy composites can be improved by adding a small amount of CNC-UPy. This reflected the good mechanical properties of PVA/CNC-UPy composite membranes. At the same time, further tests also showed that PVA/CNC-UPy/FA composite hydrogels possessed good sustained release and self-healing performance for the soil conditioner, FA.
